# Utilization of defined microbial communities enables effective evaluation of meta-genomic assemblies

**DOI:** 10.1186/s12864-017-3679-5

**Published:** 2017-04-13

**Authors:** William W. Greenwald, Niels Klitgord, Victor Seguritan, Shibu Yooseph, J. Craig Venter, Chad Garner, Karen E. Nelson, Weizhong Li

**Affiliations:** 1 Bioinformatics and Systems Biology, University of California San Diego, La Jolla, CA USA; 2 Human Longevity Inc, San Diego, CA USA; 3 J. Craig Venter Institute, La Jolla, CA USA; 4 Department of Computer Science, University of Central Florida, Orlando, FL USA

## Abstract

**Background:**

Metagenomics is the study of the microbial genomes isolated from communities found on our bodies or in our environment. By correctly determining the relation between human health and the human associated microbial communities, novel mechanisms of health and disease can be found, thus enabling the development of novel diagnostics and therapeutics. Due to the diversity of the microbial communities, strategies developed for aligning human genomes cannot be utilized, and genomes of the microbial species in the community must be assembled de novo. However, in order to obtain the best metagenomic assemblies, it is important to choose the proper assembler. Due to the rapidly evolving nature of metagenomics, new assemblers are constantly created, and the field has not yet agreed on a standardized process. Furthermore, the truth sets used to compare these methods are either too simple (computationally derived diverse communities) or complex (microbial communities of unknown composition), yielding results that are hard to interpret. In this analysis, we interrogate the strengths and weaknesses of five popular assemblers through the use of defined biological samples of known genomic composition and abundance. We assessed the performance of each assembler on their ability to reassemble genomes, call taxonomic abundances, and recreate open reading frames (ORFs).

**Results:**

We tested five metagenomic assemblers: Omega, metaSPAdes, IDBA-UD, metaVelvet and MEGAHIT on known and synthetic metagenomic data sets. MetaSPAdes excelled in diverse sets, IDBA-UD performed well all around, metaVelvet had high accuracy in high abundance organisms, and MEGAHIT was able to accurately differentiate similar organisms within a community. At the ORF level, metaSPAdes and MEGAHIT had the least number of missing ORFs within diverse and similar communities respectively.

**Conclusions:**

Depending on the metagenomics question asked, the correct assembler for the task at hand will differ. It is important to choose the appropriate assembler, and thus clearly define the biological problem of an experiment, as different assemblers will give different answers to the same question.

**Electronic supplementary material:**

The online version of this article (doi:10.1186/s12864-017-3679-5) contains supplementary material, which is available to authorized users.

## Background

Human microbiomes are the communities of microbial organisms that exist on and in our bodies, and are known to interact with our bodies in many ways. Recent studies have linked features of the microbiome to human health including brain, heart, liver and gut health [1–4]. It is thought that identifying and studying these features at both population and individual levels will provide insight into disease risk [5]. However, the complexity of the challenge is not small given that the number of distinct microbial cells are estimated to be about 1.3 times larger than that of the human host [6], and the gene content is perhaps an order of magnitude larger than that [7]. The majority of human microbiome studies have been taxonomic in nature, focusing on 16S rDNA gene sequencing and analysis, which has been useful, but fails to get at the functional differences within and between species. The recent explosion in the NGS space, which has allowed for whole genome sequencing of microbial communities [5], holds significant promise in this respect. Unlike single organism studies, a comprehensive set of genomes from which to align is not available, and de novo assembly of sequence reads into contigs is required for functional level analysis. In this regards, the field is not yet fully developed - different studies researching the microbiome utilize different analysis pipelines with different assemblers [8–12]. The assembler chosen has been shown to have an effect on the results obtained from the study [13].

The assembler used affects the length and quality of the contigs generated from a NGS run, thus impacting the conclusions drawn about a microbial sample. It is therefore important to make an informed decision when choosing an assembler for a pipeline. As new assemblers become available, it is necessary to quantify assembler quality by benchmarking them against existing tools using a consistent but relevant set of metrics. Furthermore, the samples used to benchmark these assemblers need to reflect the true complexity of a biological sample, including being defined enough to be quantifiable. Different large data sets, such as the terrestrial sediment metagenomic dataset from Sharon et al. [14], and those derived from the NIH Roadmap Human Microbiome Project (HMP) [15], have been used to benchmark the performance of assemblers. While these datasets may capture the true complexity of a human microbiome, measuring the performance and specific strengths of metagenomic assemblers on these datasets is still a challenge, as the true genomic content of these samples is not known [14, 15]. To circumvent this problem and evaluate the various aspects of assemblers more closely, we utilized communities of species with known reference genomes in known abundances. Despite the reduced complexity of these datasets when compared to most human microbiome samples, the ability to precisely test different challenges an assembler may face allows for a more in-depth analysis of each metagenomic assembler, ultimately allowing for an unbiased selection that is dependent on the task at hand.

To determine the quality of metagenomic assemblies, the microbiome community often looks at 4 (or more) metrics focused on the nucleotide contigs created by the assembler: the mean size of the contigs in the assembly, the size of the largest contig in the assembly, the number of misassemblies created by the assembler, and the length of contig, X, where the total length of all contigs of length ≥ X is greater than or equal to half of the total assembly size (N50) [8–11, 16]. These metrics give a good basis for determining the assembler’s ability to join low coverage points of the genome, as well an understanding of the assembler’s ability to distinguish between similar regions across different genomes within the metagenomic set. They fail, however, to address questions of functionality, such as the number of correctly reconstructed ORFs versus the number of de novo ORFs.

**Fig. 1:**
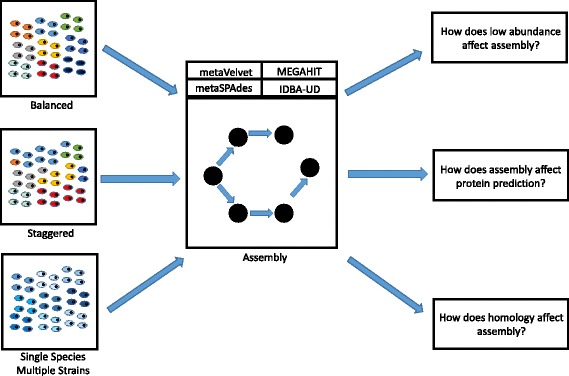
NGS reads from three different types of communities—the “balanced” community comprised of 20 unique strains of bacteria on the same order of abundance, a “staggered” community of the same 20 unique strains of bacteria with highly variable abundances, and single species communities comprised of 10 unique strains of a single species—were given as input to five different metagenomic assemblers: Omega, metaVelvet, MEGAHIT, metaSPAdes and IDBA-UD. Each assembler created contigs from the reads. By comparing the contigs generated, the ORFs called from the contigs, and the abundances of the ORFs and species, three different challenges metagenomic assemblers face were tested specifically. The results from these precise comparisons allows for a directed selection of assembler when completing a specific research goal

The tool at the forefront of measuring assembler efficacy against the previous metrics is metaQUAST [16]. However, most metagenomic data sets that assemblers have been measured against via metaQUAST contain microbial communities that are either complex and unknown, such as the HMP [15], or known but contain only a handful of species [17]. The larger data sets, while accurately mimicking the human microbiome, convolutes the challenges facing the assembler, and the smaller data sets do not contain enough diversity to challenge the assembler. In this study, we utilize the metaQUAST tool to evaluate assemblies for multiple medium sized, complex, known real and synthetic communities. Each community is designed to evaluate a different challenge a metagenomic assembler may face (Fig. 1).

As metagenomic assembly is still a work in progress, there are a plethora of metagenomic assemblers to test utilizing various algorithmic and computational approaches; the Omega [18] assembler utilizes overlap graphs, whereas MEGAHIT [10], IDBA-UD [8], metaSPAdes [9], metaVelvet [11], SOAPdeNovo2 [19], and RayMeta [20] are *de Bruijn* graph based. Furthermore, RayMeta is implemented using MPI, while other approaches run on standalone Linux system. In recent years, *de Bruijn* graph based assemblers have been successfully used to assembly next generation short reads. We picked five of the available assemblers to compare as follows: MEGAHIT was chosen as it is the successor to SOAPdeNovo2 (https://github.com/aquaskyline/SOAPdenovo2), which is used by the recently developed and popular MOCAT2 pipeline [12]; metaSPAdes as it was released recently and had self-reported strong performance; IDBA-UD due to its strong performance as noted by Nurk et. al [9]; metaVelvet, due to its reported accuracy on low abundance species; and Omega as it is an overlap graph based assembler.

We utilized the BEI resources microbial mock community (BEI #HM-783D). This community of microbes is comprised of 20 different species with known, diverse, reference genomes. BEI created two separate datasets using this community; the previously published Mock Balanced community [21], and the newly presented Mock Staggered community, which is a community comprised of the same microbes present in the BEI mock community, but at different relative abundances (Additional file 1: Table S1).

We also tested each assembler against a synthetic community comprised of multiple different strains of 4 different species: *Escherichia coli*, a highly sequenced organism with an open pan-genome; *Staphylococcus aureus*, a highly sequenced organism with a closed pan-genome; *Bacillus fragilis*, a largely benign microbe found in the gut with opportunistic pathogen potential; and *Peptoclostridium difficile*, a commonly found gut microbe with serious pathogenic potential. While it is expected that all assemblers will perform much worse with these communities, it is important to understand the ability of assemblers to create contigs unique to each strain. By comparing the efficacy of the assemblers within both of these frameworks, we are able to determine the assemblers that are strong at finding accurate contigs between and within species, as well as those that can create accurate contigs for low abundance species.

Finally, we evaluate the differences of the functional predictions from each metagenomic assembler by comparing the ORFs found in the assembly against the ORFs in the reference genome. As the microbes within a metagenomic ecosystem interact through the metabolites they consume and produce, functional abundance prediction has been suggested as an accurate indicator of health that is modulated by the microbiome [5]. By examining the effects each assembler has on nucleotide similarity via ORF similarity and abundance prediction for each community, we are able to gain a refined understanding into the choice of metagenomic assembler.

## Methods

### Mock community DNA

The following reagent was obtained through BEI Resources, NIAID, NIH as part of the Human Microbiome Project: Genomic DNA from Microbial Mock Community B (Staggered, Low Concentration), v5.2 L, for 16S rRNA Gene Sequencing, HM-783D.

### Mock communities

For the analysis of the BEI balanced mock community, we utilized the data previously generated as described in our previous study [21]. Similarly, the DNA for staggered mock communities were generated as described with input concentrations of DNA per organism are as found in Additional file 1: Table S2. Library preparation and sequencing were done utilizing the sample protocol as described in the same paper [21].

### Synthetic communities

Ten strains of each of *Staphylococcus aureus*, *Bacillus fragilis* and *Peptoclostridium difficile*, were arbitrarily chosen and the verified unique reference genome sequences for the strain were pooled into a single reference file per species.

### Read simulation

Reads were simulated for the single species communities via wgsim (https://github.com/lh3/wgsim). The community was simulated at a uniform coverage per strain determined by the length of the strain’s genome. Otherwise, the −1 100, −2 100 and -d 300 flags were set.

### Genomes used in simulations

The full genome sequences for read simulation were downloaded from NCBI. A full list of strain taxonomy identifiers and accession numbers for all organisms can be found in Additional file 1: Table S3.

### Assembly

Prior to assembly, raw paired end reads were filtered using Trimmomatic [22] (option: SLIDINGWINDOW:4:15 LEADING:3 TRAILING:3 MINLEN:90 MAXINFO:80:0.5). This trims the reads using a sliding window of size of 4 with average quality score <15. After trimming, if either read R1 or R2 is shorter than 90 bases, the whole read pair is considered low quality and is removed from further analysis. After applying sequence quality filters, the balanced and staggered Mock communities had 15,468,061 and 13,557,702 high quality paired end reads, respectively, that were used as inputs for all the assemblers. Each dataset was assembled using: metaSPAdes version 3.8.1 with default parameters except for –meta and --only-assembler. The choice of kmer was managed by metaSPAdes program; it creates graphs with 3 different kmer lengths. IDBA-UD 1.1.2 was run with --mink = 50 --maxk = 80 --step = 10, and --min_contig = 180. The choice of kmer of 50–80 with step 10 for IDBA-UD is based on our previous analysis that reached optimal performance. After sequence quality filtering and trimming, reads of at least 90 bases were kept. We thus did not use a kmer of 90 or longer. Shorter kmers (k = 30, 40), were not used as they performed much worse than the longer kmers. MEGAHIT1.0.6 was run via “--presets meta”, as the program manual suggested. MEGAHIT also automatically uses multiple kmers in graph construction. The metaVelvet 1.2.01 pipeline was first running velveth 51 -fasta -shortPaired, then velvetg -exp_cov auto -ins_length 300; and finally meta-velvetg -ins_length 300 in standard, non-SL mode, installed with MAXKMERLENGTH = 63, as the velvet pipeline suggested this MAXKMERLENGTH when run with default parameters. Omega was run with -l 60 as suggested for our insert size by the Omega manual.

### Assembled reads estimation

Not all programs used for analysis reported the number of assembled reads. We thus estimate the number of assembled reads by aligning the reads to the contigs of each assembler via bwa [23] mem with default parameters.

### ORF calling

To find the ORFs present on each contig, the program Metagene [24] was used with default parameters on the contig set generated from each assembler.

### ORF clustering

To determine reference/aberrant ORFs, cd-hit [25] was used with -c 0.95 -n 5 -M 1600 -d 0 and -T 8 to cluster the combined set of ORFs from contigs and ORFs from reference genomes. Clusters of reference-only ORFs were called missing, and clusters of contig-only ORFs were called aberrant. The contig ORFs in the remaining clusters with reference ORFs are considered correct ORFs.

### Aligning reads to contigs

To align the reads to the generated contigs, bwa mem was used with default settings. The output SAM file was subsequently filtered to keep only the top hit (s) for each read.

### Abundance prediction

Reads were assembled into contigs as described above from which ORFs were then predicted using metagene as described above. Similarly, ORFs were predicted from the known references for the real samples. The combined set of ORFs were clustered with cd-hit as described above. All clusters containing a single reference ORF were kept. The original reads were aligned to the contigs and samtools mpileup was then run to find the number of bases at each position for each contig. The abundance for each organism is then calculated as the average coverage for each ORF that was in a cluster containing a reference ORF for that organism.

## Results

A majority of microbial communities are heterogeneous in composition as well as abundance. Failure to accurately reconstruct the genomes of low abundance organisms within the community is of concern as these errors could miss critical functions that pertain to the disease and health of the community or host. To assess the ability of assemblers to recover low abundance species, we contrast the performance of each assembler on balanced and staggered communities with the same organisms. The major performance indicators, including largest contig, number of misassemblies, fraction of genome coverage, number of contigs and N50, are highlighted in Tables 1 and 2 and are discussed in the following paragraphs.

**Table 1:** Statistics from assembly of the mock balanced community

Assembly	metaSPAdes	IDBA-UD	metaVelvet	MEGAHIT	Omega
Largest contig	1072791	886024	453604	566646	1304789
# misassemblies	61	58	21	42	310
Genome fraction (%)	98.724	98.291	97.45	99.033	97.664
Number contigs	1875	3245	6892	2979	3051
N50	109515	60486	23780	66228	87111

**Table 2:** Statistics from assembly of the mock staggered community

Assembly	metaSPAdes	IDBA-UD	metaVelvet	MEGAHIT	Omega
Largest contig	413864	413271	244874	376020	381247
# misassemblies	66	71	5	80	120
Genome fraction (%)	62.318	57.563	33.978	60.826	47.344
Number contigs	10577	12499	3787	11305	10198
N50	15191	9903	24705	12028	24944

### Balanced community

**Fig. 2:**
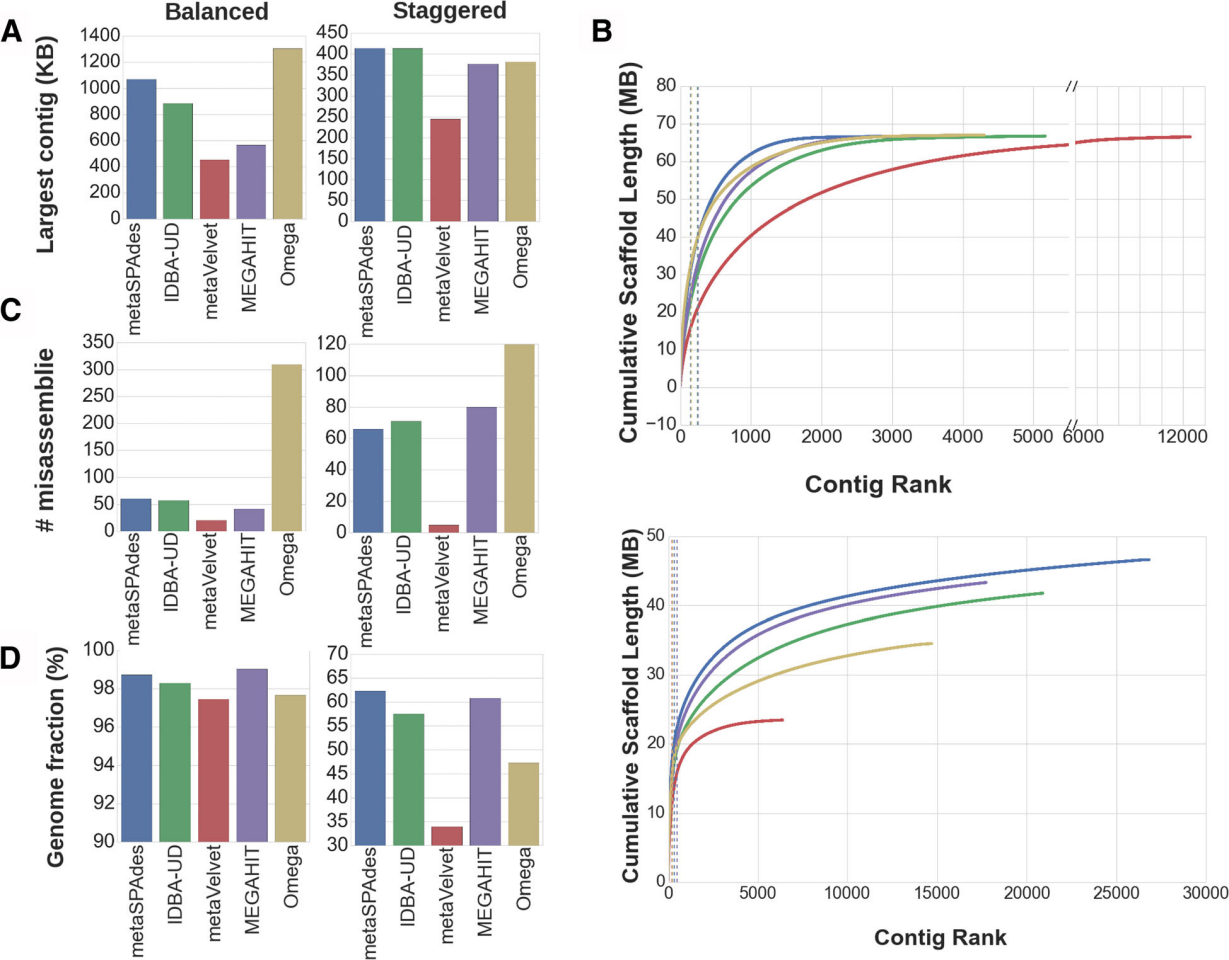
Assembler performance was measured in both communities by (**a**) largest contig. Contigs were ranked by length and are plotted against cumulative length of assembly for balanced (**b** *top*) and staggered (**b** *bottom*).   Furthermore, assembly performance was measured in both communities by (**c**) the number of misassemblies and (**d**) PRG assembled. *Dashed lines* represent the contig number of the N50, with N50 occurring at the intersection of the *curve* and it’s *dashed line*

Within the balanced community, Omega was able to assemble the largest contig, followed by metaSPAdes, IDBA-UD, MEGAHIT and finally metaVelvet (Fig. 2a). The total length of all assemblies from the balanced community were within 1 MB of the same size of one another (Fig. 2b), though metaVelvet assembles more contigs to reach its total assembly length. Furthermore, the N50 for metaSPAdes is noticeably (20-40 kb) larger than those from Omega, IDBA-UD and MEGAHIT, all of which are also 40–60 kb larger than metaVelvet (Fig. 2b and Table 1). However, when examining the number of misassemblies created by each assembler, the pattern is reversed—metaVelvet has the least number of misassemblies, followed by MEGAHIT, IDBA-UD, metaSPAdes and finally Omega (Fig. 2c), though Omega makes 5–14 fold more errors than any of the other assemblers. MetaQUAST reports the percent of the reference genome (PRG) covered from the concatenated genomes of all reference organisms in the mock community. For this metric, we see a third pattern—MEGAHIT covers the most, then metaSPAdes, then IDBA-UD, then Omega and finally metaVelvet, though all are within 2 percentage points of one another (Fig. 2d). All assemblers were estimated to utilize at least 99.0% of input reads during assembly, in concordance with their near complete PRG.

### Staggered community

When examining the staggered community, the differences in the largest contig size from metaSPAdes, IDBA-UD and MEGAHIT remains quite small—Omega has a greater largest contig size, and metaVelvet has a much smaller largest contig size (Fig. 2a). The total length of the assemblies, however, are quite different (Fig. 2b). MetaSPAdes covers the most number of bases, followed by MEGAHIT, then IDBA-UD, then Omega and finally metaVelvet. The N50s of the staggered community are also different from the balanced community, with metaVelvet and Omega almost 10 kb larger than metaSPAdes, followed by MEGAHIT and finally IDBA-UD (Fig. 2b and Table 2). It is important to note that while the N50 is much larger for metaVelvet and Omega, the number of bases in the assemblies are much smaller than the others. MetaSPAdes, IDBA-UD and MEGAHIT assembled over 99.0% of the input reads, Omega assembled 98.1% of the input reads, and metaVelvet assembled 94.8% of the input reads. As most reads in the staggered community are from high abundance, and thus well assembled, organisms, it is anticipated that a high fraction of reads are assembled. It is important to note, however, that two assemblers can assemble the same number of reads, yet capture organisms at different abundances, as one assembler could utilize a large quantity of reads from high abundance organisms, and another could utilize a large quantity reads from low abundance organisms. The difference in assembler performance is thus better compared through the PRG of each assembly: metaSPAdes covers the most, followed by MEGAHIT, IDBA-UD, Omega and finally metaVelvet (Fig. 2d). The differences in PRG are concordant with with the abundance of the species within the staggered community. Furthermore, there is a large difference between the number of misassemblies from each assembler, perhaps due to the large disparity in the number of bases covered by metaVelvet versus the other assemblers, and the difference in assembly graph traversal approach in Omega: metaVelvet has the least with 5, followed by metaSPAdes at 66, IDBA-UD at 71, MEGAHIT at 80 and Omega at 120 (Fig. 2c).

### Synthetic communities

**Fig. 3:**
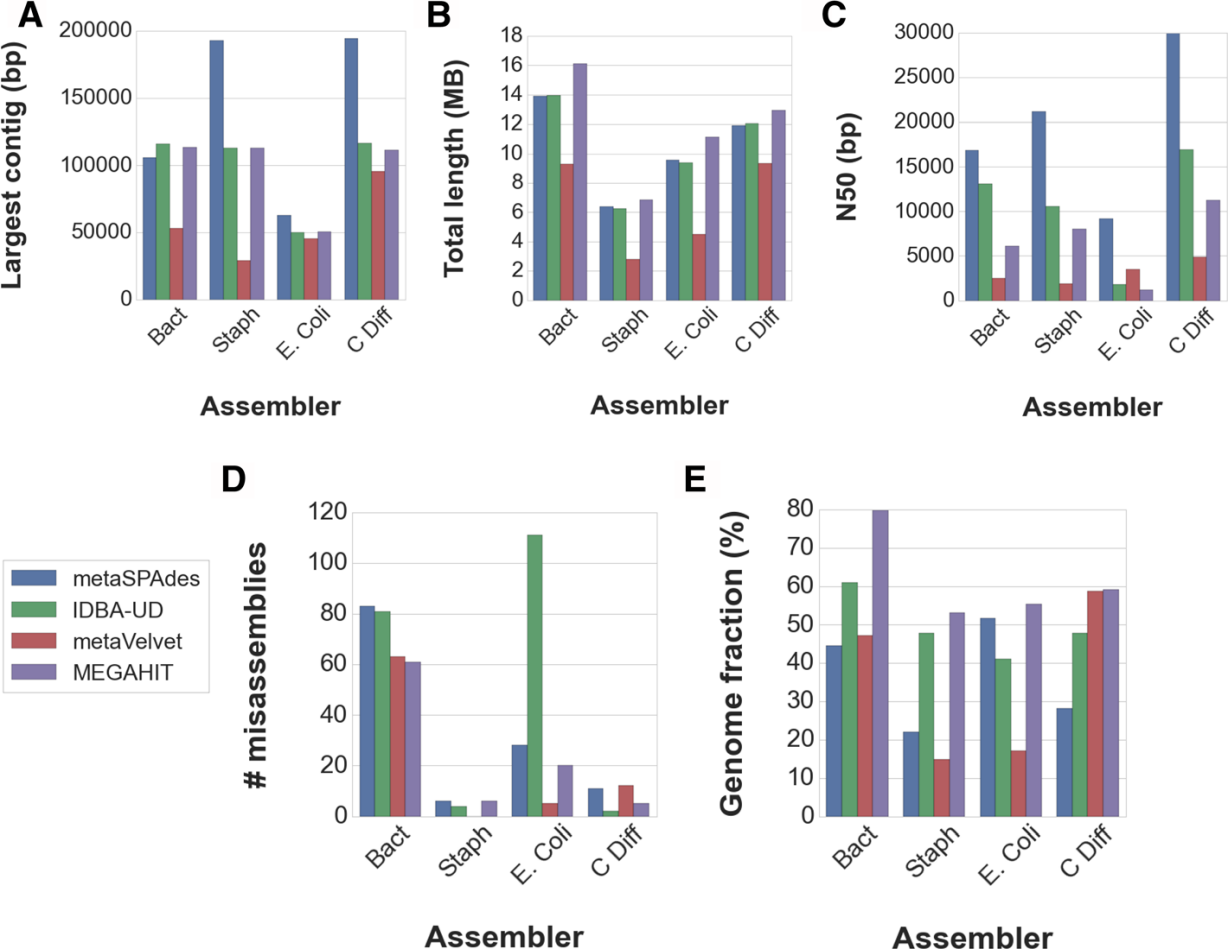
Assembler performance measured by **a** largest contig, **b** total assembly size in megabases, **c** N50, **d** number of misassemblies and **e** PRG shown for each assembler on simulated microbial communities from 10 strains of B. fragilis, S. aureus, E. Coli and P. difficil

To determine the efficacy of each assembler to accurately reconstruct strains, we simulated four unique balanced communities of multiple strains from the same species. We did not include Omega in further analyses due to the larger error rate in both mock communities, and small PRG from the staggered community (indicating a loss of information). For three of the four single species communities, metaSPAdes has the largest contig (Fig. 3a), and for the *B. fragilis* community, metaSPAdes, IDBA-UD and MEGAHIT all have similar largest contig sizes (Fig. 3a). MEGAHIT has the largest assembly for each community, closely followed by metaSPAdes and IDBA-UD (Fig. 3b). MetaSPAdes has a much larger N50 than the other assemblers for the communities (Fig. 3c). The number of misassemblies per community is close across assemblers, except for *E. coli* reads assembled with IDBA-UD, which has four fold more misassemblies as the next greatest assembler, metaSPAdes (Fig. 3d). Finally, the genomic fraction covered by each assembly is much smaller than that of the mock community, which is between 10 and 80% smaller depending on the assembler and the community. MEGAHIT has the most for three of the four communities, and is on par with metaVelvet for the *P. difficile* community. However, metaVelvet either had the lowest PRG, or was within 3% of the next lowest PRG, for the other three communities (Fig. 3e).

### ORF prediction

**Fig. 4:**
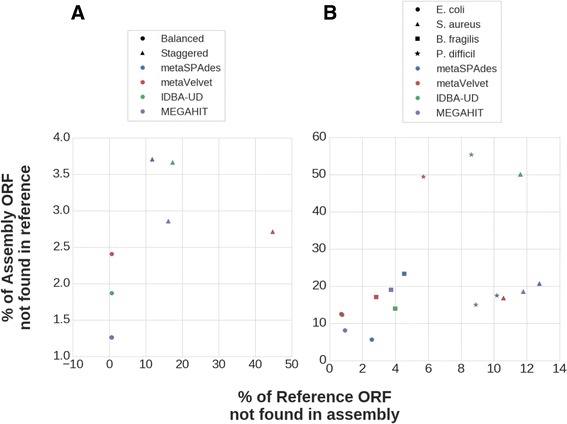
Percent of ORFs present in the joint reference but missing from the assembly (missing) vs percent of ORFs present in the contigs but missing from the reference set (aberrant) for the BEI mock communities (**a**) and the single species communities (**b**). An even trade off would be visualized as  a linear shift across the assemblers. A decrease in missing ORFs is usually paired with an increase in aberrant ORFs, with a larger magnitude of change in the number of aberrant ORFs. Points in the single species community cluster more closely by species (*shape*) than by assembler (*color*), indicating that the similarity in the sequences being assembled play a larger role in misassemblies than the assemblers themselves—general patterns within assemblers are still present

The contigs generated by assemblers may not accurately recapitulate the ORFs from the reference genome, either by missing ORFs or creating novel incorrect (aberrant) ORFs. Within the balanced community, all assemblers are able to recall over 99% of the ORFs from the reference set, however, the assemblers have differing levels of aberrant ORF calls, with metaVelvet having the most followed by IDBA-UD, and MEGAHIT and metaSPAdes having roughly the same (Fig. 4a). The staggered community, however, is extremely variable between the different assemblers. MetaSPAdes has the least amount of missing ORFs, and metaVelvet has an extremely high number of missing ORFs, whereas metaVelvet has the least number of aberrant ORFs and metaSPAdes has the most (Fig. 4a).

The single species communities cause a high level of variance in the performance of the various assemblers. For the *E. coli* and *P. difficile* communities, IDBA-UD and metaVelvet predict a far larger number of aberrant ORFs than either MEGAHIT or metaSPAdes (Figs. 4b and 3d). However, MEGAHIT and metaSPAdes both have a larger number of missing reference ORFs than metaVelvet or IDBA-UD. In the *S. aureus* community, IDBA-UD has over double the number of aberrant ORF than the other assemblers, whereas other metrics are close to one another (Fig. 4b). Finally, in the *B. fragilis* community, IDBA-UD has the least number of Aberrant ORFs, but the relative difference between the best and worst assembler is much less than in the other communities (Fig. 4b).

**Fig. 5:**
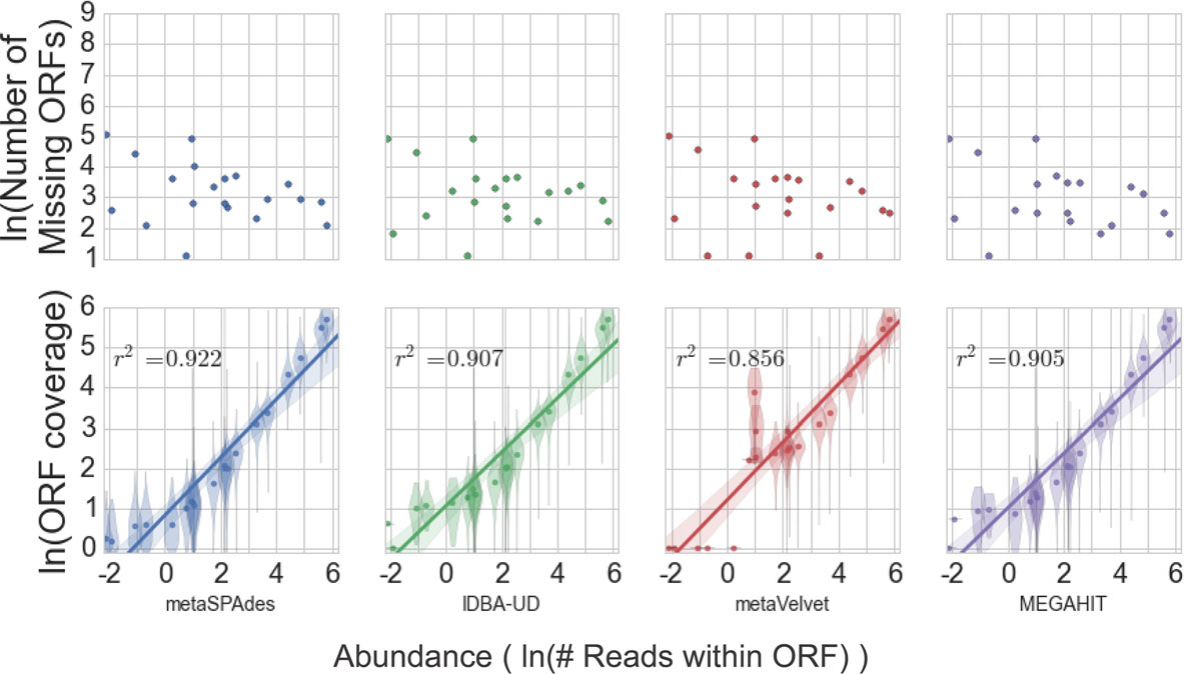
Concordance of species coverage predicted by reads (x-axis, both plots) with species coverage predicted by ORFs (*bottom*) and concordance of total missing ORFs with abundance of species (*top*) for the Staggered community for each assembler. Both sets of graphs are plotted on natural log vs natural log scales. For regression between coverages, mean values were used—violins of the ORF coverage distributions are shown surrounding each point

It is difficult to examine the accuracy of the abundance of each ORF in the community due to the diversity of proteins in the community, and the difficulty of measuring individual protein concentrations. Thus, as a proxy for accuracy of ORF abundances, we examined the concordance of species abundances from reads mapping to the reference with species abundances from ORF abundances. All assemblers have similar estimates and the same coefficient of determination (COD, *R* ^*2*^ = 0.99) for the abundances of microbes within the balanced community (Additional file 2: Figure S1, top). Staggered abundance prediction, however, varies, with metaSPAdes having the strongest COD (*R* ^*2*^ = 0.922) with the true relative abundances, followed by MEGAHIT (*R* ^*2*^ = 0.905), IDBA-UD (*R* ^*2*^ = 0.907), and finally metaVelvet (*R* ^*2*^ = 0.856) (Fig. 5b). It is important to note that the number of ORFs found for some species is much lower in comparison to metaSPAdes. IDBA-UD and MEGAHIT both only found a single ORF from one low abundance species, whereas metaSPAdes has no singleton species. Furthermore, IDBA-UD and MEGAHIT both miss one species in their abundance estimates, and metaVelvet misses 4 more in addition to the aforementioned 1.

## Discussion

The quality of a metagenomic assembly is assessed by its similarity to the reference set at either the nucleotide or protein level. As metagenomic assemblies often contain multiple contigs for the same reference, unique sets of nucleotide level contigs do not necessarily contain the same protein information, as ORF prediction can be disrupted at the edges of a contig. We thus aim to assess the assemblers in both contexts, as different metagenomics experiments may desire accuracy for different information.

The real mock communities were utilized to test the ability of an assembler to find low abundance species, while having an equal abundance community present as a control. Species missing from both communities do not necessarily indicate a difficulty in estimating the abundance of scarce organisms, but rather a difficulty in assembling the sequence for the species.

Within the balanced community, all assemblers covered a similar number of bases and PRG. However, while Omega, metaSPAdes and IDBA-UD had much larger longest contigs, MEGAHIT has the most stable contig size, evidenced by its N50 almost equaling its largest contig, and large linear range (Fig. 2b). While there is no clear choice for the best assembler for nucleotide level information for the balanced community as metaVelvet had a much lower number of misassemblies than the other community despite having lower scores in the other metrics (Fig. 2), Omega makes 5–14 fold more errors than the other assemblers, making its output uninformative.

To understand the ability of each assembler to identify low abundance organisms, we compared the staggered community performance to the balanced community performance. When shifting from assembling the balanced community to the staggered community, metaSPAdes has a comparable number of misassemblies (Fig. 2c), a 600 KB shorter longest contig (Fig. 2a), 20 M less bases in its assembly (Fig. 2b) and 30% less PRG (Fig. 2d). IDBA-UD also had a performance drop when used on the staggered community, though it was less of a drop than metaSPAdes; IDBA-UD’s longest contig fell by 400 kb (Fig. 2a), but had a larger increase in misassemblies than metaSPAdes (Fig. 2c). In comparison to IDBA-UD, metaSPAdes appears to capture longer and more accurate nucleotide level information. It is also important to note that MEGAHIT has a large PRG (on par with metaSPAdes) despite its short longest contig and smaller N50 than metaSPAdes.

In the balanced communities, MEGAHIT and metaVelvet both have shorter longest contigs than metaSPAdes and IDBA-UD (Fig. 2a), but cover a comparable number of nucleotides in their assemblies (Fig. 2b). The shift to assembling a staggered community causes MEGAHIT to make the second most misassemblies of all assemblers tested (Fig. 2c). However, MEGAHIT’s PRG is similar to that of metaSPAdes (Fig. 2d). Despite metaVelvet having the smallest contigs, its N50 remains unchanged by the staggered community (Fig. 2b). By examining the correlation of PRG with true species abundance, we are able to see that metaSPAdes and MEGAHIT are capturing species across all abundances, while IDBA-UD misses a few at low abundance.

MetaVelvet, on the other hand, has a lower PRG yet detects species in low abundance well (Figs. 2d and 5), indicating that it is missing information from abundant species. The number of misassemblies for metaVelvet decreases when shifting to the staggered community as well. There are two possible explanations: metaVelvet is skipping lowly abundant species, thus not capturing their sequence and reproducing the same errors as in the balanced community; or metaVelvet is missing the low-abundant species and thus not incorporating them into chimeric contigs, thereby both missing some sequence data and skipping chimeric contigs as compared to the balanced community. Regardless of the cause, metaVelvet captures the most accurate nucleotide level information for scarce species, albeit in small chunks. A tool that combines both metaVelvet and metaSPAdes may result in the longest and most accurate contigs for low-abundant species.

In a separate pattern from the other four assemblers, Omega has the largest longest contig size in both the balanced to the staggered communities (Fig. 2a), yet a PRG in-between that of metaVelvet and the other assemblers (Fig. 2d). Furthermore, the number of misassemblies in Omega remains far above (5–24×) the others in both the balanced and staggered communities (Fig. 2c). The low PRG combined with the high number of missassemblies, large contig size and large size of misassembled contigs (Additional file 1: Tables S4 and S5) indicates that Omega is potentially over scaffolding, similar to metaSPAdes, yet only capturing a small amount of the population, similar to metaVelvet. This combination indicates that Omega captures a small, yet highly erroneous, portion of the community. The high number of errors may be due to the overlap graph approach of Omega.

To understand how well assemblers can delineate strains of the same species, synthetic communities of multiple strains from the same species of microbes in balanced abundance were simulated. Unsurprisingly, the assemblers did not perform as well on these communities than the previous mock communities (Fig. 3). While metaSPAdes continued to have the largest contigs and N50, MEGAHIT consistently had the largest assembly size and largest PRG. The number of misassemblies appears to depend more on the species being assembled than the assembler being used since the number of misassemblies per community is close across assemblers, except for *E. coli* with IDBA-UD, which created 4 times as many misassemblies as metaSPAdes. Thus, MEGAHIT is an excellent choice for recovering the different serotypes within a microbial community. MEGAHIT, for example, would be ideal for detecting a particular pathogen in a community of similar but non-pathogenic species.

To evaluate the effect of the breakpoints between contigs generated by the assemblers on protein abundance prediction, we used MetaGene to call ORFs from assembled contigs. The only reads used for assembly were those that came from the reference genome, therefore, only two types of ORFs can be predicted by MetaGene: 1) ORFs from the reference data set that were assembled correctly; or 2) aberrant ORFs, which are not present in the reference. These ORFs are the only possibilities as it is not possible for an ORF that is absent from the reference to be a novel and correct ORF since the BEI mock community is comprised of organisms with known complete references, and the single species communities were simulated data sets.

In the balanced community, the assemblers can recapitulate over 99% of the reference ORFs, and only vary by 1% for aberrant ORFs; the staggered community, however, has a larger disparity (Fig. 4a). Overall, as points shift to the right on the X-axis, they also shift down the Y-axis, indicating a relationship between the number of aberrant ORFs and the number of missed reference ORFs. The increase in the number of aberrant ORFs, however, is much larger than the number of missing reference ORFs.

While metaVelvet creates accurate contigs (Fig. 3d), the number of breakpoints within the contigs causes a large loss of reference ORFs from the data set. MetaVelvet does, however, creates the smallest number of aberrant ORFs. MetaSPAdes has the least number of missing reference ORFs, and the most number of aberrant ORFs. This relationship is complementary to our previous notion that metaVelvet, while having a much smaller amount of the metagenomic data set covered by its contigs, has a much higher quality in the assembly for low-abundant microbes. Similarly, metaSPAdes, while capturing the most information, is highly prone to making mistake in low abundance organisms during its scaffolding process.

The trade-off of a larger change in the number of aberrant ORFs created than the number of reference ORFs found is apparent in the single species communities as well (Fig. 4b), though the ordering of accuracy within assemblers is shifted. Notably, the organism being assembled has a much larger role in the capability of an assembler to accurately assemble ORFs than the assembler itself. Despite the large role species plays in assembler accuracy across all communities, metaSPAdes consistently misses the largest number of ORFs from the reference, and metaVelvet captures the highest number of ORFs from the reference. IDBA-UD had a large change depending on the community, having the lowest number of aberrant ORFs for *B. fragilis*, but the largest by a wide margin for *S. aureus*. Over all communities, MEGAHIT is consistently in the middle or the lowest, furthering its prowess for strongly related community assembly.

We also assessed how sensitive each assembler was to the relative abundance of the organisms present in its ability to successfully reconstruct the expected ORFs. This analysis was done by comparing the relative abundance of each species relative to the absolute number of missing ORFs from that species for each assembler (Fig. 5, top). MetaSPAdes is the most linear with its drop in performance with low abundant species, whereas MEGAHIT and IDBA-UD both have a large, quick drop at mid abundance. MetaVelvet has a bimodal distribution, with a large number of missing ORFs at low abundance, and then almost no missing ORFs at high abundance. These results further metaSPAdes as a strong choice for ORF prediction in diverse communities where important functions might only be found at low abundances, while also suggesting that metaVelvet might be appropriate for ORF prediction in the case where one favors accurate information for the most prevalent functions in the community.

Finally, some efforts have examined functional capabilities of a community as a whole. It is extremely difficulty and infeasible, however, to accurately measure a community’s protein abundances for ORF abundance comparison. We thus used a proxy to measure how each assembler distorts the true abundances of ORFs. To do so, we used the concordance of species coverage measured by mapping reads to the joint reference genomes with the average coverage of ORFs called by the assembler for each species. All assemblers recapitulate the mock balanced community to relatively the same abundances, with identical CODs (*r* ^*2*^ = 0.99, Additional file 2: Figure S1, bottom). We expect this similarity due to the similarity between all previous metrics examined for the balanced community.

There is a small difference, however, between an assembler’s ability to determine the relative abundances of species within the staggered community. This difference mirrors the ability of each assembler to recreate reference ORFs. MetaSPAdes is able to most accurately reproduce the relative abundances compared to IDBA-UD, MEGAHIT or metaVelvet (Fig. 5, top). Though the difference in the COD is quite small for metaSPAdes, IDBA-UD and MEGAHIT, metaSPAdes has more normally distributed ORF coverage profiles at the lower abundances than IDBA-UD and MEGAHIT, indicating it is finds a more consistent abundance across the ORFs it reassembles. Furthermore, it misses no species, while MEGAHIT and IDBA-UD each miss one, and only call a single ORF for another (Fig. 5, bottom).

## Conclusions

Depending on the metagenomic task, different assemblers should be chosen. Prior knowledge about the diversity and relative abundances of the data set allows for an informed choice of assembler. Within low abundance environments, metaVelvet makes the smallest assemblies, but has a very small number of misassemblies within those contigs. MetaSPAdes has the highest number of misassemblies, but creates the longest contigs. Because of this, metaSPAdes is an excellent choice for determining ORFs within a metagenomic sample.

Within communities with similar microbes, MEGAHIT does an excellent job of reconstructing different contigs from the set. Furthermore, it does well at recreating the functional abundance profile of a community. IDBA-UD, while not leading in any category, does not preform the worst in any category, lending itself as a great metagenomic assembler for nucleotide level information when prior information about the community is not known. Future algorithms that combine the results from multiple assemblers could provide higher quality and longer contigs by preferring sequences generated by metaVelvet, and incorporating them into the calls by metaSPAdes or MEGAHIT depending on diversity and abundance of the microbes within the target metagenomic ecosystem. As metaVelvet captures highly accurate information, revising the matching contigs or parts of scaffolds from metaSPAdes and MEGAHIT to match metaVelvet may help resolve some misassemblies created. A simple default to the metaVelvet contigs for similar sequences may not be complex enough to capture the diversity and low abundance species that metaSPAdes, MEGAHIT and IDBA-UD find, however. A tool which creates a consensus assembly from the combination of metaVelvet and either MEGAHIT or metaSPAdes may prove to find the most accurate information.

## Additional files

Additional file 1: Table S1. Species abundance in mock staggered community. **Table S2.** DNA concentration in mock staggered community. **Table S3.** Tax ID and Accession Numbers for all organisms in mock or synthetic communities. **Table S4.** MetaQUAST output from mock balanced community. **Table S5.** metaQUAST output from mock staggered community. (XLSX 84 kb)

Additional file 2: **Figure S1.** Concordance of species coverage predicted by reads (x-axis, both plots) with species coverage predicted by ORFs (y-axis) (bottom) and concordance of total missing ORFs (y-axis) with abundance of species (top) for the Balanced community for each assembler. Both sets of graphs are plotted on natural log vs natural log scales. For regression between coverages, mean values were used—violins of the ORF coverage distributions are shown surrounding each point. (PDF 104 kb)

## Abbreviations

COD: Coefficient of determination
HMP: Human Microbiome Project
MB: Megabase
NGS: Next generation sequencing
NIH: National Institutes of Health
ORF: Open reading frame
PRG: Percent of reference genome
