# Supplementary figures and images for: Utilization of defined microbial communities enables effective evaluation of meta-genomic assemblies
Source: BMC Genomics. 2017 Apr 13;18:296. doi: 10.1186/s12864-017-3679-5 (PMC5390407; doi:10.1186/s12864-017-3679-5)

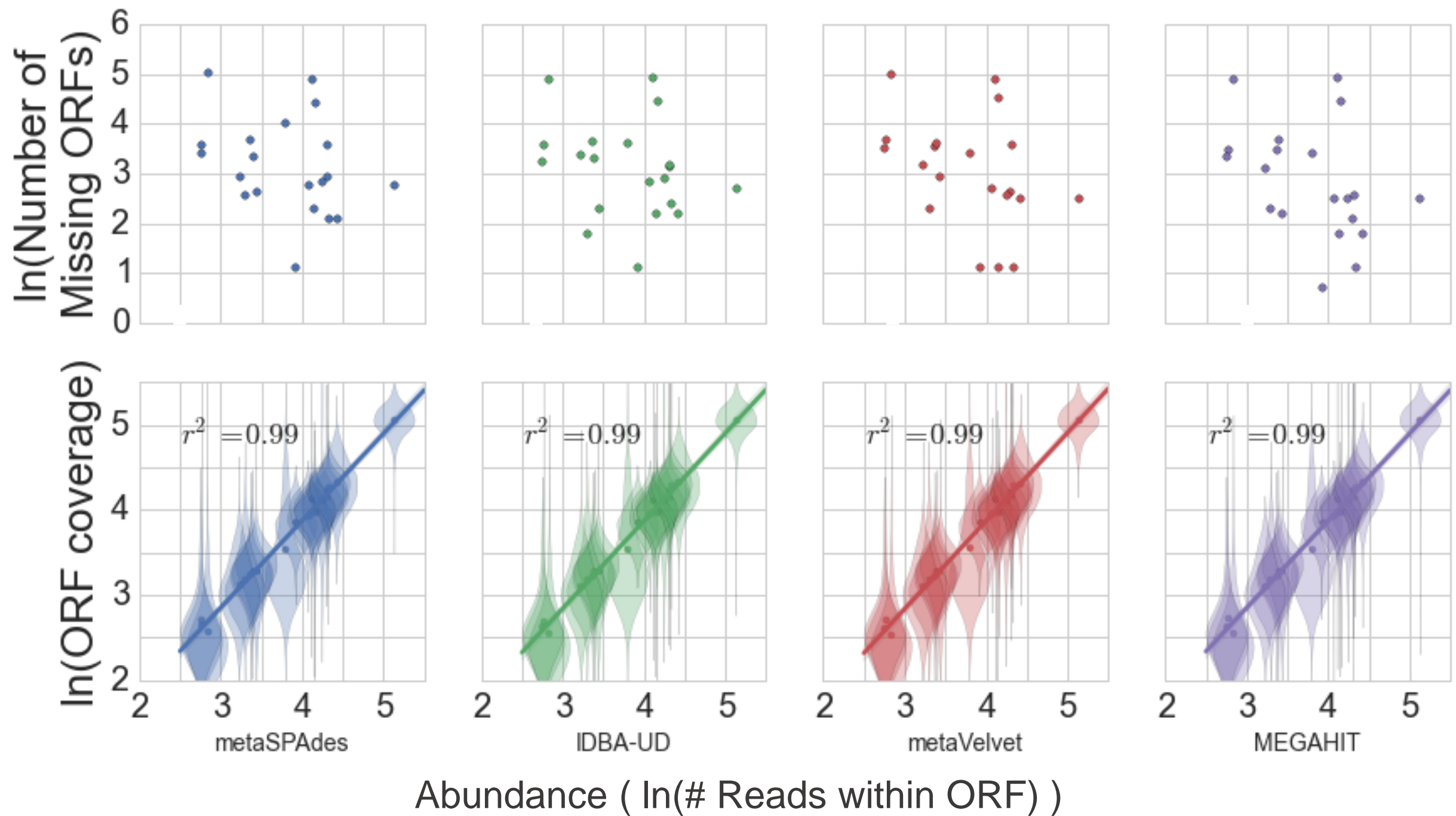

Supplement: Supplementary file 2 — Figure S1. Concordance of species coverage predicted by reads (x-axis, both plots) with species coverage predicted by ORFs (y-axis) (bottom) and concordance of total missing ORFs (y-axis) with abundance of species (top) for the Balanced community for each assembler. Both sets of graphs are plotted on natural log vs natural log scales. For regression between coverages, mean values were used—violins of the ORF coverage distributions are shown surrounding each point. (PDF 104 kb) [file 12864_2017_3679_MOESM2_ESM.pdf]
